# Proper Care of Infants

**Published:** 1893-05

**Authors:** S. T. Rober


### PROPER CARE OF INFANTS.

Of the total number of deaths in warm weather, according to the records, nearly one-third are children under one year of age. Now, is this mortality a necessary evil? We are inclined to think not. The diseases from which these children die arise, as a rule, from preventable causes. Not always so much from the want of care as from the want of knowledge how to care for them. The device of any means to prevent this great infant mortality is a work well worthy of a philanthropist. If we look to the classes of disease closely, the inference is that a considerable number of these deaths have in reality a close and direct relation to the kind of food given.

The general cry of teething is nonsensical. If a child is bathed and fed regularly, clothed loosely and comfortably, not over nursed or mauled on a warm day and given regular hours of rest, the teeth will come through almost unnoticed. It must be remembered that a child cannot digest food containing starch, such as rice water, bread food, pap or gruel, until it has teeth. Therefore milk, which is animal food has by nature been given as the only one needed up to that time.

Careful examinations prove that the highest mortality is among children that are brought up by hand. This shows for itself that they are given a pure substitute for their natural food. Cow's milk, slightly watered and sweetened with sugar of milk, is perhaps one of the best substitutes for mother's milk; although the writer has had excellent results from some of the well known infants' foods. A tablespoonful of lime water may be added to each pint of milk occasionally.

It is a most sorrowful sight to behold a haggard, restless, moaning child huddled up in warm arms, or heavily blanketed on a feather pillow in a baby coach on a hot July day. And very, very often a long tubed bottle is in the mouth, which perhaps has been there for one or two hours. This is enough to kill the child, if nothing more. Mothers, let me tell you that most of these troubles can be avoided. Take your babe from its bed every morning at a regular hour, bathe it well, but carefully, in lukewarm salt water; dry with a soft towel. If it is discolored by heat dust it lightly with rice flour, then put next to its body an all wool gauze flannel shirt long enough to cover its abdomen, over this a thin flannel shirt with muslin or linen body; then its slip, which should be simple and plain, with high neck and long sleeves. Zephyr socks should cover its feet, and a linen bib protect the neck of the dress. After this feed, if not from the breast, prepare a half-pint of milk, turn it into a sweet bottle and put on a short nursing tube. Hold the child when feeding in a semi-erect position. If the child is on its back or side the milk is likely to be thrown up and lost to the child. How often we see a nurse in feeding a child by the bottle fix them comfortably in bed on their backs, put the tube in their mouth and leave them to suck as much milk and air into their stomachs as they conveniently can. All this favors those accidents which it is desirable to avoid. After the child has taken the half-pint of milk lay it down on a mattress, cover lightly, and allow it to take a good, long nap, which in most cases it will gladly do.

After this nap a four or five months' baby should be fed regularly every three hours, and should not under any circumstances be fed oftener. Overfeeding frequently produces the same result as starvation. The child should have another rest at about 1 o'clock. At the close of the day, say 6 o'clock, undress it, rub lightly with the hand and change all its clothing for a night suit, which may be about the same as the one taken off. Never allow the child to sleep in garments worn during the day. After this, feed and put it to bed. It is a great mistake to keep a child out in the night air in a coach, disturbing it to transfer it to bed at a later hour. When disturbed in this way, the child may fret all night. No food is required between 11 and 5, unless the child be awake from some unnatural cause. A flannel bag, 4x5 inches, containing a teaspoonful of ground cloves, a teaspoonful of cinnamon, a half-teaspoonful of ginger, a teaspoonful of allspice, a grated nutmeg and three or four blades of bruised meadow mint, should be made and kept in the house to use if necessary. This bag, moistened with alcohol, made warm and placed over the stomach will allay vomiting, or over the abdomen will soothe and govern the bowels. It can be used as long as the spices retain their strength. S. T. ROBER, in an exchange.

### SLEEP.

There is no fact more clearly established in the physiology of man than this, that the brain expends its energies and itself during the hours of wakefulness, and that these are recuperated during sleep; if the recuperation does not equal the expenditure, the brain withers. This is insanity.

Thus it is that in early English history, persons who were condemned to death by being prevented from sleeping always died raving maniacs. Thus it is, also, that those who are starved to death become insane; the brain is not nourished and they cannot sleep. The practical inferences are three: 1st, those who think most, who do most brain work, require most sleep; 2d, that time "saved" from necessary sleep is infallibly destructive to mind, body and estate; 3d, give yourself, your children, your servants, give all who are under you the fullest amount of sleep they will take, by compelling them to go to bed at some regular early hour, and to rise in the morning the moment they *awake of themselves*; and within a fortnight nature, with almost the regularity of the rising sun, will unloose the bonds of sleep the moment enough repose has been secured for the wants of the system.

This is the only safe and sufficient rule; and as to the question, *how much sleep any one requires*, each must be a rule for himself. Great nature will never fail to write it out to the observer, under the regulations just given.

